# Reviewer acknowledgement 2014

**DOI:** 10.1186/s12861-015-0052-2

**Published:** 2015-01-21

**Authors:** Philippa K Harris

**Affiliations:** BioMed Central, 236 Gray’s Inn Road, London, WC1X 8HB UK

## Abstract

The editors of *BMC Developmental Biology* would like to thank all our reviewers who have contributed to the journal in Volume 14 (2014).

**Craig Albertson**

USA

**Declan W. Ali**

Canada

**Jeffrey Amack**

USA

**Ana Angelova Volponi**

UK

**Christopher Antos**

Germany

**Michalis Averof**

France

**Alfred Batschauer**

Germany

**Nathalie Beaujean**

France

**Mark Berryman**

USA

**Marco Biggiogera**

Italy

**Samuel Breit**

Australia

**Cathrin Brisken**

Switzerland

**Aaron Brown**

USA

**Thomas Carroll**

USA

**Francesc Cebria**

Spain

**Sang-Wook Cha**

USA

**Tatiana Chebotareva**

UK

**Steven Cheng**

China

**Rosanna Chianese**

Italy

**Ariel Chipman**

Israel

**Barry Condron**

USA

**Frank Conlon**

USA

Stephanie Cossette

**USA**

**Selahattin Danisman**

Germany

**Fiorenza De Bernardi**

Italy

**Katia Del Rio-Tsonis**

USA

**Franck Delaunay**

France

**Alison Dell**

USA

**Herman Dierick**

USA

**Virginia Diewert**

Canada

**Wolfgang Driever**

Germany

**Richard M. Epand**

Canada

**Maria-José Escribá**

Spain

**Alexei Evsikov**

USA

**Paul Fowler**

UK

**Tamara Franz-Odendaal**

Canada

**Brian Gabrielli**

Australia

**Gary Gaufo**

USA

**Burhan Gharaibeh**

USA

**Roman Giger**

USA

**Sarah Goetz**

USA

**Kaoru Goto**

Japan

**Mukesh Kumar Gupta**

India

**Marnie Halpern**

USA

**Andreas Heyland**

Canada

**Andreas Hiltbrunner**

Germany

**Seiji Hitoshi**

Japan

**Katja Hummitzsch**

Australia

**Helen Irving-Rodgers**

Australia

**Jr Jacobs**

Canada

**Anna Jazwinska**

Switzerland

**Yasuhiko Kawakami**

USA

**Nobuyuki Kawashima**

Japan

**Wiliam Kelly**

USA

**Khandan Keyomarsi**

USA

**Toshikazu Kondo**

Japan

**Ping Kong**

USA

**Laijun Lai**

USA

**Tilman Lamparter**

Germany

**Pierre Leclerc**

Canada

**Armand Leroi**

UK

**Michael Levin**

USA

**Gil Levkowitz**

Israel

**Baojie Li**

China

**Lingyu Li**

USA

**Ziyi Li**

China

**Ching-Ling (Ellen) Lien**

USA

**Gufa Lin**

USA

**Susana Lopes**

Portugal

**Scott Lozanoff**

USA

**P Mabee**

USA

**Paul Maddox**

USA

**Fanny Mann**

France

**Julio Martin**

Spain

**Maria D Martin-Bermudo**

Spain

**Chris Mayhew**

USA

**Dies Meijer**

UK

**Myrte Merkestein**

UK

**Anthony Mescher**

USA

**Mark Metzstein**

USA

**Silvia Clotilde Modina**

Italy

**Denise Montell**

USA

**Bernard Moussian**

Germany

**David Murphy**

UK

**Christopher Navara**

USA

**Xavier Nicol**

France

**Heiner Niemann**

Germany

**Hirotaka James Okano**

Japan

**Katsuhiko Ono**

Japan

**Christopher Ormandy**

Australia

**Achim Paululat**

Germany

**Christopher Payne**

USA

**Natalie Payne**

Australia

**Francisco Pelegri**

USA

**Roberta Pennati**

Italy

**Christian Petersen**

USA

**M.Dolors Piulachs**

Spain

**Berenika Anna Plusa**

UK

**Ken Prehoda**

USA

**Don Ready**

USA

**Joao Relvas**

Portugal

**Rene Rezsohazy**

Belgium

**Sandra Rieger**

USA

**Filippo Rijli**

Switzerland

**Paul Riley**

UK

**Rakel Romar**

Spain

**Siegfried Roth**

Germany

**Charles Sagerstrom**

USA

**Jean-Luc Scemama**

USA

**Sylvie Schneider-Maunoury**

France

**Norito Shibata**

Japan

**Fraser Sim**

USA

**Johan Smitz**

Belgium

**Josefa Steinhauer**

USA

**Scott Stewart**

USA

**Michael Stock**

Germany

**Padraig Strappe**

Australia

**Devi Suman**

USA

**Genlou Sun**

Canada

**Beat Suter**

Switzerland

**Tohru Suzuki**

Japan

**Piroska Szabo**

USA

**Qinghua Tao**

China

**Kevin Temeyer**

USA

**X Cindy Tian**

USA

**Simon Tuck**

Sweden

**Alexander Vaiserman**

Ukraine

**Michael Todd Valerius**

USA

**Srinivasan Vijayaraghavan**

USA

**Xin Wang**

USA

**Timothy Weil**

UK

**Gunther Wennemuth**

Germany

**Gary Wessel**

USA

**Gabrielle Wheway**

UK

**Gregory Wray**

USA

**Chun-Fang Wu**

USA

**Xuechun Xia**

China

**Ting Xie**

USA

**Yukiko Yamazaki**

USA

**Gyesoon Yoon**

South Korea

**Xin Yuan**

USA

**Vincenzo Zappavigna**

Italy

**Meijia Zhang**

China

